# Redirecting Monocyte Differentiation With Engineered Extracellular Vesicles for Glioma Immunotherapy

**DOI:** 10.1002/advs.76910

**Published:** 2026-08-05

**Authors:** Yuanwei Pan, Xuan Liu, Qian‐Fang Meng, Lei Cao, Rongrong Li, Yangtao Xu, Jing Zhang, Chenchen Zhao, Lang Rao, Chen Yu

**Affiliations:** ^1^ Institute of Chemical Biology Shenzhen Bay Laboratory Shenzhen China; ^2^ Institute of Cancer Research Shenzhen Bay Laboratory Shenzhen China

**Keywords:** cancer immunotherapy, dual targeting, extracellular vesicles, glioma, monocyte‐derived macrophage

## Abstract

During glioma progression, monocytes abundantly infiltrate but primarily differentiate into immunosuppressive macrophages to promote tumor growth. Redirecting monocyte differentiation offers a compelling yet underexplored therapeutic opportunity. In this work, we found M1‐polarized macrophage‐derived extracellular vesicles (M1‐EVs) efficiently induced monocytes to differentiate into anti‐tumor macrophages via tumor necrosis factor alpha (TNF‐α)‐mediated signaling. Despite promising, the therapeutic efficacy of M1‐EVs was constrained by insufficient glioma accumulation and CD47‐mediated phagocytic inhibition. To address this challenge, we further engineered M1‐EVs with dual‐targeting specificity by genetically incorporating a tumor‐directed chimeric antigen receptor (CAR) against IL13Rα2 or EGFRvIII together with CD47‐blocking SIRPα variants. The resulting dual‐targeting EVs (M1‐CS‐EVs) exhibited enhanced blood‐brain barrier (BBB) penetration and glioma accumulation while locally disrupting CD47‐SIRPα interactions. In three orthotopic glioma models, M1‐CS‐EVs elicited a potent anti‐tumor immune response and enhanced tumor phagocytosis, significantly suppressing tumor growth while prolonging animal survival. Our findings establish a platform technology for directing monocyte differentiation toward anti‐tumor phenotypes, offering a broadly applicable strategy for glioma treatment.

## Graphical abstract

A dual‐targeting engineered extracellular vesicles (M1‐CS‐EVs) platform is developed to redirect monocytes differentiation into anti‐tumor macrophages for glioma immunotherapy. This nanoplatform combines CAR‐mediated tumor recognition with localized CD47 blockade, leading to synergistic immune activation and potent suppression of tumor progression in orthotopic glioma models.

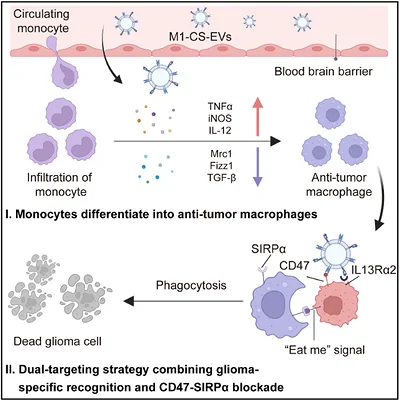

## 1 Introduction

Glioma is the most common and aggressive primary brain tumor, with a dismal prognosis despite advances in surgery, radiation, and chemotherapy [1, 2]. Immunotherapy poses a promising treatment option for cancer [3]. However, the benefit of immunotherapy for glioma remains suboptimal due to the unique brain immune profiles and immunosuppressive tumor microenvironment (TME) [4, 5, 6, 7]. A critical limitation is the poor infiltration and impaired function of T cells, which are consistently sparse in glioma across both early and late stages of tumor progression, rendering T cell immunotherapy for glioma largely ineffective [8, 9, 10, 11]. In contrast to the limited T cell presence, monocyte infiltration into the TME is robust and increases significantly with tumor progression [12, 13]. Circulating monocytes are recruited to the brain in response to inflammatory signals and differentiate into monocyte‐derived macrophages (MDMs) [14, 15]. However, these MDMs primarily adopt an immunosuppressive pro‐tumor phenotype [16], aiding tumor immune evasion by inhibiting antigen presentation and T cell proliferation [17], thus promoting an immunosuppressive TME [18]. Monocytes retain substantial plasticity and can be redirected toward anti‐tumor effector functions upon appropriate stimulation [19, 20, 21]. Thus, redirecting monocytes differentiation during glioma progression offers a compelling yet underexplored therapeutic opportunity.

Monocyte‐to‐macrophage differentiation is a critical process in immune modulation, with various strategies explored to direct polarization toward anti‐tumor phenotypes [22, 23, 24, 25, 26]. Cytokine‐based therapies such as granulocyte macrophage colony‐stimulating factor (GM‐CSF) can effectively polarize monocytes toward anti‐tumor macrophages [27, 28, 29], but their clinical application in glioma is limited by poor blood‐brain barrier (BBB) penetration and systemic inflammatory side effects [30, 31, 32, 33]. While mRNA delivery systems offer improved durability in monocyte programming [34], their efficacy in glioma remains constrained by inefficient intracranial delivery and potential off‐target immune activation [35, 36, 37]. Emerging evidence suggests that extracellular vesicles (EVs) may overcome some of these limitations due to their natural biocompatibility and BBB penetration, as well as their ability to participate in intercellular communication [38, 39]. Recent studies have demonstrated that M1‐polarized macrophage‐derived EVs (M1‐EVs) inherit proinflammatory cytokine contents including tumor necrosis factor 𝛼 (TNF𝛼) and interleukin 6 (IL‐6), which can polarize macrophages toward an anti‐tumor phenotype, suggesting their capacity to modulate immune cell behavior [40, 41, 42]. Importantly, these cytokines are known to regulate monocyte activation through post‐transcriptional regulation of inflammatory mediators [43], suggesting that M1‐EVs have the potential to orchestrate monocyte‐to‐macrophage differentiation through immediate cytokine signaling [44, 45, 46]. Although M1‐EVs have shown the potential to differentiate monocytes into anti‐tumor macrophages, their therapeutic efficacy in gliomas is ultimately compromised by the interaction of glioma‐overexpressed CD47 with macrophage SIRPα, which acts as a “don't eat me” signal that suppresses phagocytic activity [47].

The CD47‐SIRPα signaling axis serves as a critical immune checkpoint that inhibits phagocytic activity of macrophages derived from infiltrating monocytes in gliomas [48], where CD47 overexpression correlates with immune evasion [49]. Although CD47 blockade via anti‐CD47 antibodies or SIRPα‐Fc fusion proteins can augment macrophage activity and decrease phagocytosis resistance [50], systemic administration risks anemia and thrombocytopenia due to ubiquitous CD47 expression on normal cells [51]. Moreover, single‐targeting approaches face fundamental limitations imposed by the dynamic glioma microenvironment, including heterogeneous target expression and compensatory immune evasion mechanisms that often lead to treatment failure [52, 53, 54, 55]. This underscores the necessity for strategies that can spatially restrict CD47 inhibition to the TME while simultaneously ensuring precise delivery to glioma cells [56, 57]. EVs engineered with chimeric antigen receptor (CAR) constructs present an optimal solution, they inherit the ability to cross the BBB while gaining tumor‐specific targeting through CAR expression [58, 59, 60, 61]. By further equipping these CAR‐EVs with high‐affinity SIRPα variants, they can achieve glioma‐specific accumulation through CAR‐mediated recognition, and localized disruption of CD47 signaling via membrane‐anchored SIRPα, thereby unleashing macrophage phagocytosis without systemic toxicity [62, 63]. This integrated dual‐targeting approach addresses the key limitations of current strategies by combining precision targeting with localized immune checkpoint modulation.

Herein, we present a promising strategy by harnessing the intrinsic BBB‐traversing capability and immunomodulatory potency of M1‐EVs to induce differentiation of circulating monocytes into anti‐tumor macrophages, subverting the immunosuppressive glioma microenvironment. Single‐cell transcriptomics revealed that M1‐EVs drive this differentiation primarily through TNF‐α‐mediated pathway activation, generating immunostimulatory macrophage populations with distinct anti‐tumor signatures. To amplify therapeutic effects through dual‐targeting engagement, we engineered these M1‐EVs to co‐express multiplexed CARs targeting glioma‐associated antigens IL13Rα2 or EGFRvIII, along with a high‐affinity SIRPα variant blocking the CD47‐SIRPα pathway (M1‐CS‐EVs). In orthotopic glioma models, M1‐CS‐EVs demonstrated superior tumor accumulation than non‐targeting EVs and triggered potent phagocytosis of glioma cells. The combined action of CAR‐mediated tumor targeting and localized CD47 blockade resulted in significant tumor volume reduction and survival extension. By integrating EV‐based monocyte differentiation with spatially controlled CD47 inhibition and tumor‐specific targeting, we demonstrate a promising direction for glioma immunotherapy that overcomes the immune evasion and delivery challenges that limit current approaches.

## 2 Results

### 2.1 M1‐EVs Induce Monocytes to Differentiate Into Anti‐Tumor Macrophages

Glioma progression is characterized by persistently low T cell infiltration but a marked expansion of pro‐tumor macrophages [4]. This immunologic signature was evident in the Cancer Genome Atlas (TCGA) datasets, which revealed no significant difference in CD8^+^ T cell infiltration between healthy and glioma samples, but a clear enrichment of pro‐tumor macrophages in glioma (Figure 1A,B). This finding was further confirmed by analysis of the tumor immune microenvironment, which showed that immune cells, especially macrophages and monocytes, were almost exclusively concentrated within the tumor region, with negligible infiltration in healthy brains or peri‐tumoral regions (Figure 1C–E). Immunofluorescence staining confirmed the immunosuppressive phenotype of glioma‐infiltrating macrophages, with high expression of markers associated with pro‐tumor functions (Figure 1F). The strong correlation between pro‐tumor macrophage and monocyte infiltration, along with the well‐established role of monocytes as macrophage precursors [8], suggests that continual monocyte recruitment and subsequent differentiation represent a major source of pro‐tumor macrophages in glioma.

**FIGURE 1:**
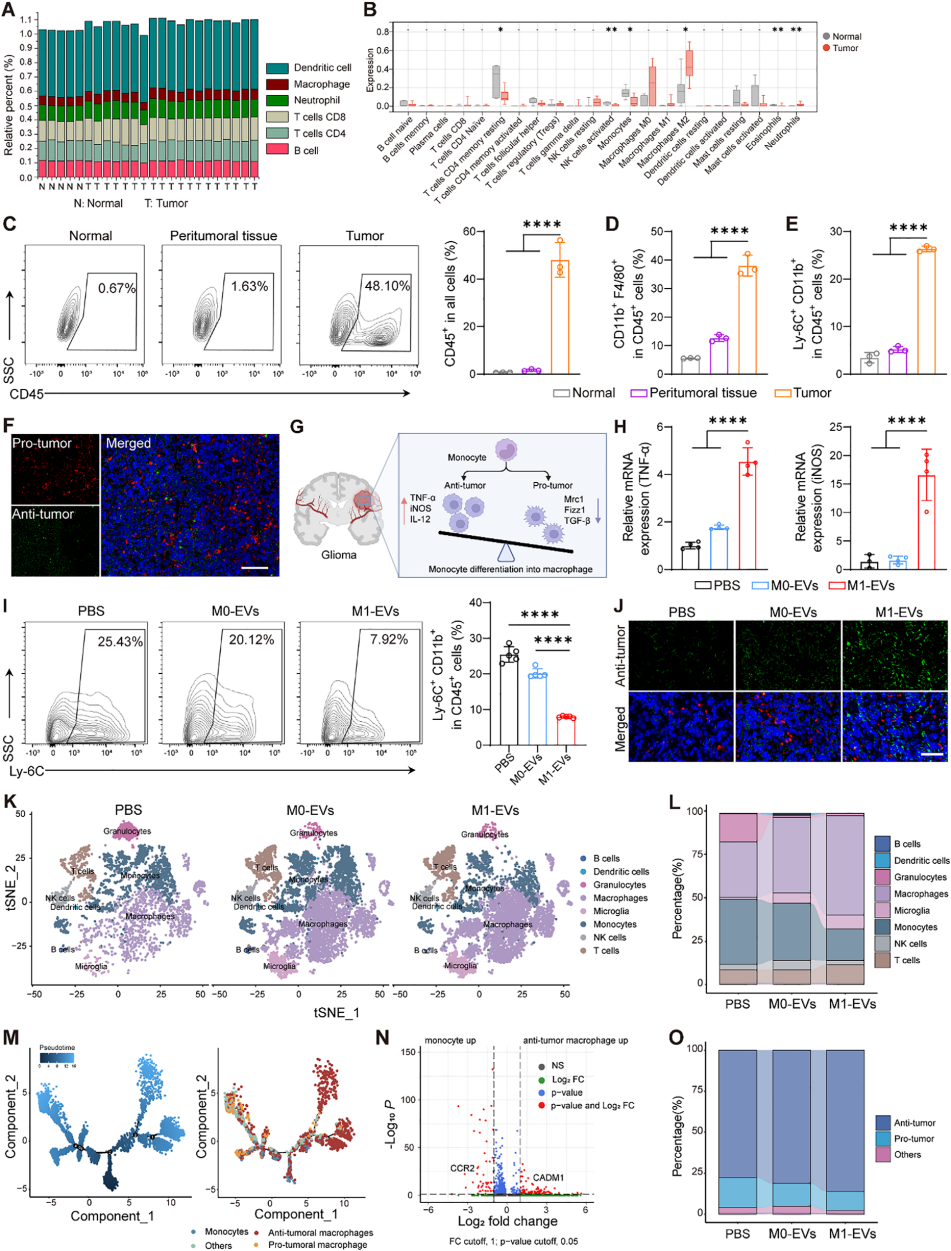
M1‐EVs induce monocytes to differentiate into anti‐tumor macrophages. (A) Histograms of the infiltration of 6 different specific immune cells in normal and tumor patients by the CIBERSORT method. (B) Differences in 22 different specific immune cell expression values between normal and tumor patients in TCGA database. (C–E) Flow cytometric analysis of immune cells (CD45^+^) (C), macrophages (F4/80^+^CD11b^+^CD45^+^) (D), and monocyte (Ly‐6C^+^CD11b^+^CD45^+^), (E) in normal mice tissue, peritumoral tissue, and tumor tissues (*n* = 3). (F) Immunofluorescence analysis of anti‐tumor macrophages (CD86^+^) and pro‐tumor macrophages (CD206^+^) in GL261‐IL13Rα2 tumor‐bearing mice. Scale bar, 50 µm. (G) Schematic diagram of M1‐EVs inducing monocytes to differentiate into anti‐tumor macrophages. (H) Relative expressions of mRNA in anti‐tumor markers (TNF‐𝛼 and iNOS) as evaluated by qPCR after intravenous (*i.v*.) injection with PBS, M0‐EVs or M1‐EVs (*n* = 4). (I) Flow cytometric analysis of monocyte (Ly‐6C^+^) in tumor tissues gating on CD11b^+^CD45^+^ cells after the indicated treatments (*n* = 5). (J) Immunofluorescence analysis of anti‐tumor macrophages (CD86^+^) and pro‐tumor macrophages (CD206^+^, red) in GL261‐IL13Rα2 tumor‐bearing mice after the indicated treatments. Scale bar, 40 µm. (K) t‐SNE map of CD45^+^ cell clusters from tumor‐bearing brain after different treatments. (L) Relative proportions of different types of immune cells in CD45^+^ cells. (M) Pseudotime ordering of monocyte and macrophage. (N) Volcano plot showing genes that are differentially expressed between monocyte and anti‐tumor macrophage. (O) Relative proportions of anti‐tumor and pro‐tumor macrophage. Statistical analysis was performed by one‐way ANOVA with Tukey's multiple comparisons tests. The experimental data were presented as mean ± S.E.M. ^*^ *p* < 0.05, ^**^ *p* < 0.01, ^***^ *p* < 0.001, ^****^ *p* < 0.0001.

The extensive monocyte infiltration in gliomas prompted us to explore strategies for redirecting monocyte differentiation toward anti‐tumor phenotypes [21]. Given that M1‐polarized macrophages secrete pro‐inflammatory mediators known to counteract immunosuppression, we hypothesized that their extracellular vesicles (M1‐EVs) could serve as natural vehicles for monocyte activation. We isolated M1‐EVs and found that they were enriched in mRNAs for pro‐inflammatory factors, including TNF‐α and iNOS (Figure S1A). To explore the effects of M1‐EVs on monocytes, the extracted primary mouse bone marrow‐derived monocytes were incubated with M0‐EVs or M1‐EVs. Flow cytometry analysis showed that M1‐EVs promoted the differentiation of primary monocytes into macrophages with a tumor‐suppressive phenotype in vitro (Figure 1G and Figure S1B–D). The monocyte differentiation activity of M1‑EVs is primarily mediated by TNF‑α protein, with a minor contribution from RNA carried by M1‑EVs (Figure S1E). The q‐PCR results showed that M1‐EVs enhanced the secretion of pro‐inflammatory cytokines TNF‐α, iNOS, and IL‐6, while suppressing anti‐inflammatory mediators Mrc1, Fizz1, and IL‐10 (Figure 1H and Figure S1F). This effect was recapitulated in glioma models (Figure 1I,J, and Figure S1G–J), demonstrating that M1‐EVs drove monocyte differentiation into anti‐tumor macrophages. Beyond their effects on monocyte, M1‐EVs also polarized resident microglia toward an anti‐tumor phenotype (Figure S1K). Furthermore, M1‐EVs also remodeled the glioma microenvironment to promote monocyte recruitment, as conditioned media from M1‐EV‐treated glioma cells enhanced monocyte migration in a transwell chemotaxis assay (Figure S1L).

To gain insight into how M1‐EVs drive monocyte‐to‐macrophage differentiation, we performed scRNA‐seq of glioma‐infiltrating immune cells, identifying distinct clusters (B cells, dendritic cells, granulocytes, macrophages, microglia, monocytes, NK cells, T cells) via marker genes (Figure 1K and Figures S2 and S3). Quantitative analysis revealed that M1‐EVs treatment significantly altered monocyte, granulocyte, microglia, and macrophage populations compared to PBS/M0‐EVs controls (Figure 1L), with a significant increase of macrophages and a decrease of monocytes. Pathway analysis highlighted enhanced TNF‐α response and antigen presentation but suppressed TGF‐β signaling (Figure S4). Pseudotime trajectory analysis mapped a differentiation continuum from monocytes to anti‐tumor macrophages (Figure 1M), further validated by subcluster profiling showing elevated CD68, NOS2, and CD40 but reduced CCL24 in M1‐EVs‐treated tumors (Figure S5). Volcano plot analysis revealed that TNF‐α is a central upstream regulator driving monocyte‐to‐macrophage differentiation, with CADM1, a TNF‐α‐responsive adhesion molecule, significantly upregulated, whereas CCR2, a monocyte retention receptor, was downregulated in the M1‐EV‐treated group (Figure 1N). Quantitative annotation confirmed the increase in anti‐tumor macrophage subsets after M1‐EVs treatment (Figure 1O). Collectively, these single‐cell analyses demonstrated that M1‐EVs induce TNF‐α‐dependent monocyte differentiation into anti‐tumor macrophage, effectively promoting a tumor‐suppressive immune phenotype.

### 2.2 Preparation and Characterization of M1‐CS‐EVs

Despite M1‐EVs effectively induce monocytes to differentiate into anti‐tumor macrophages, their therapeutic efficacy in glioma remains suboptimal (Figure S6A–C). To address this critical limitation, a dual‐targeting EV platform (M1‐CS‐EVs) that combines monocyte differentiation with localized CD47‐SIRPα blockade was developed (Figure 2A,B). To generate M1‐CS‐EVs, lentiviral plasmids encoding a glioma antigen‐specific CAR against IL13Rα2 paired with a SIRPα variant to block CD47 signaling were first constructed (Figure S6D), followed by macrophage transduction (Figure 2C). The expression of CAR and SIRPα variant on the corresponding cells were verified by confocal microscopy, western blotting, and flow cytometry (Figure 2D–F). The CS‐EVs isolated from macrophages inherited CAR/SIRPα proteins (Figure 2G) and expressed fluorescent markers (Figure 2H), along with EV markers CD63 and Alix. A proteinase K digestion assay confirmed that CAR and SIRPα are displayed on the external surface of CS‐EVs (Figure S6E). TEM and cryo‐EM revealed typical EV morphology (Figure 2I,J), while NTA showed homogeneous size distribution (peak: 120 nm; Figure 2K). M1‐CS‐EVs exhibited consistent size (Figure 2L) and zeta potential (Figure 2M) with control EVs (M1‐C‐EVs, M0‐CS‐EVs), demonstrating manufacturing reproducibility. Stability assessments confirmed minimal size variation over 8 days (Figure S6F), and TEM imaging further verified that the EVs maintained their typical morphology without aggregation or rupture after 8 days of storage (Figure S6G). Moreover, serum stability assays showed that M1‐CS‐EVs maintained stable size and zeta potential after 48 h incubation in 10% serum (Figure S6H). SDS‐PAGE showed comparable protein profiles across EV subtypes (Figure S7A). Collectively, these data validate the successful generation of stable, dual‐targeting M1‐CS‐EVs for targeted glioma therapy.

**FIGURE 2:**
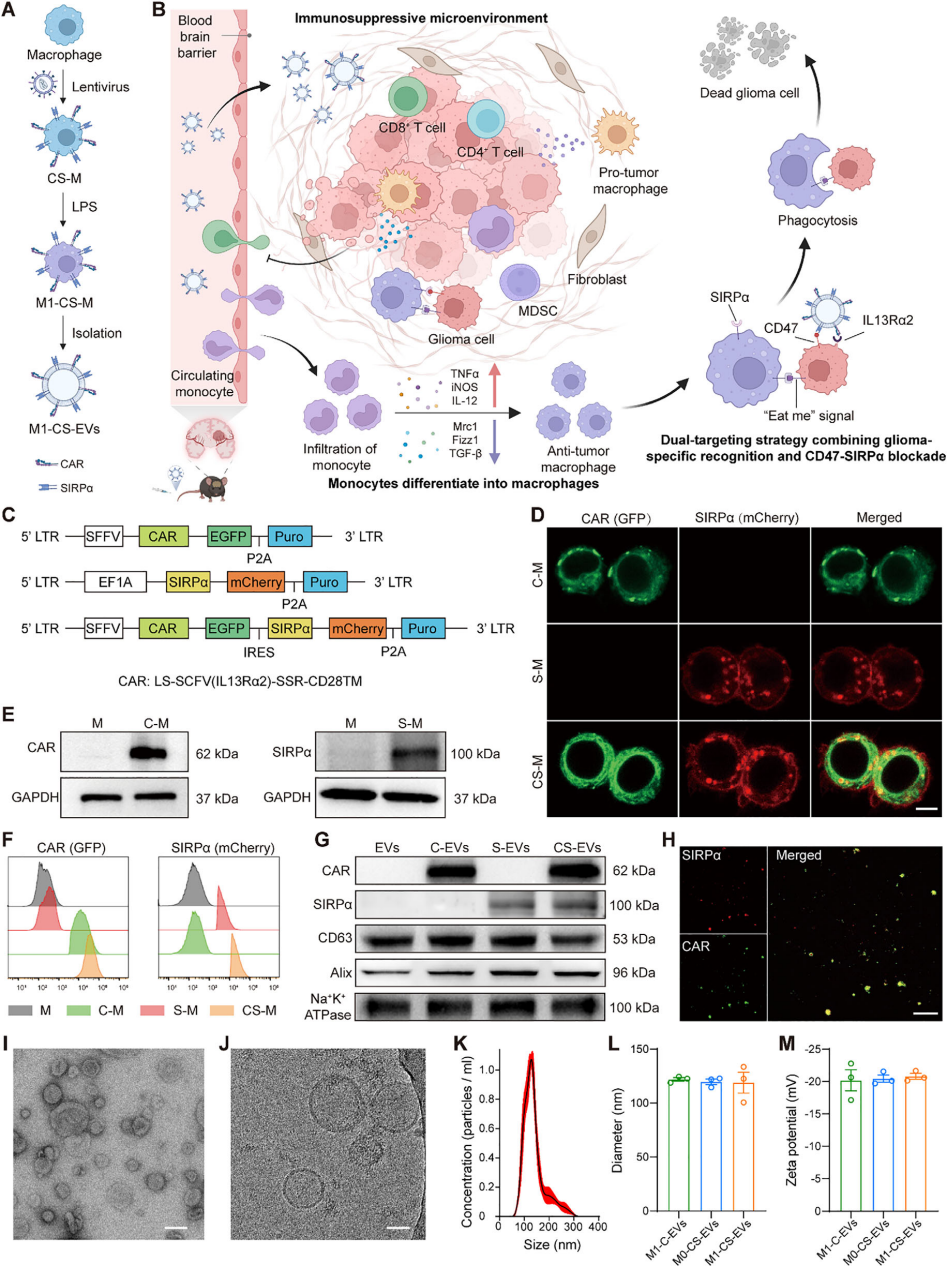
Schematic illustration and characterization of M1‐CS‐EVs. (A) The synthesis of M1‐CS‐EVs. (B) Illustration of M1‐CS‐EVs crossing the BBB, targeting tumor cells, and activating anti‐tumor immunity to combat glioma. (C) Schematic diagram of the CAR and SIRPα plasmids construction process of M1‐CS‐EVs. (D) Immunofluorescence imaging of CAR (GFP) and SIRPα (mCherry) expression on CAR‐macrophage (C‐M), SIRPα‐macrophage (S‐M), and CAR and SIRPα co‐overexpressing macrophage (CS‐M). Scale bar, 5 µm. (E) Western blotting analysis of CAR and SIRPα in M, C‐M, and S‐M. (F) Flow cytometry analysis of CAR (GFP) and SIRPα (mCherry) expression in macrophage (M), C‐M, S‐M, and CS‐M. (G) Western blotting analysis of CAR, SIRPα, CD63, Alix, and Na^+^K^+^ ATPase in various EVs. (H) Immunofluorescence images of CS‐EVs. Scale bar, 10 µm. (I) TEM image of EVs. Scale bar, 100 nm. (J) Cryo‐EM image of EVs. Scale bar, 50 nm. (K) NTA curve of EVs. (L, M) Size (L) and zeta potential (M) of M1‐C‐EVs, M0‐CS‐EVs, and M1‐CS‐EVs detected by DLS. The experimental data were presented as mean ± S.E.M. (*n* = 3).

Prior to evaluating the therapeutic functionality of our engineered EVs, their biocompatibility was first systematically assessed through comprehensive in vitro and in vivo studies. Comprehensive in vitro biocompatibility assessment revealed negligible cytotoxicity of EVs (0–100 µg mL^−^^1^) across macrophages, T lymphocytes, dendritic cells (DCs), and glioma cells (Figure S7B). To further evaluate potential acute systemic inflammation, we measured serum TNF‐α and IL‐6 levels at 4 h post‐injection in mice treated with PBS or M1‐EVs. No significant elevation of either cytokine was observed in the M1‐EVs group compared to PBS controls (Figure S7C). In vivo systemic evaluation in healthy mice demonstrated preserved tissue architecture in major organs (H&E staining; Figure S7D) and stable hematological parameters (serum biochemistry, complete blood counts; Figures S7E and S8) four weeks post‐intravenous (i.v.) administration of PBS, native EVs, or engineered EVs (M1‐C‐EVs, M0‐CS‐EVs, M1‐CS‐EVs), further confirming the favorable biosafety profile of our EV platform.

### 2.3 BBB Penetration and Glioma‐Targeted Accumulation of C‐EVs

To evaluate BBB penetration, an in vitro BBB model was established using Transwell plates (Figure 3A). DiD‐labeled macrophage‐derived EVs exhibited significantly greater translocation efficiency compared to liposomes (Figure 3B,C). Enhanced EV uptake in glioma cells was mediated by chemokine receptor interactions, as CCR2 blockade reduced cellular internalization (Figure 3D). In 3D tumor spheroids, EVs penetrated to depths of 50 µm (Figure 3E), demonstrating superior tumor penetration capabilities. In vivo studies using orthotopic GL261‐luc glioma models showed robust tumor accumulation of EVs, as evidenced by colocalization of bioluminescence (tumor) and fluorescence (EVs) signals, confirming their ability to cross the BBB and target tumor sites (Figure 3F). Time‐course analysis showed peak accumulation at 12 h post‐injection, with sustained retention for 24 h (Figure 3G,H). These findings were also confirmed and extended by ex vivo imaging of brain dissected from mice (Figure 3I). Importantly, the distribution of EVs showed minimal overlap with CD31 vasculature (Figure 3J), confirming efficient extravasation and deep tumor penetration. These findings establish EVs as effective carriers for crossing biological barriers and targeting intracranial tumors.

**FIGURE 3:**
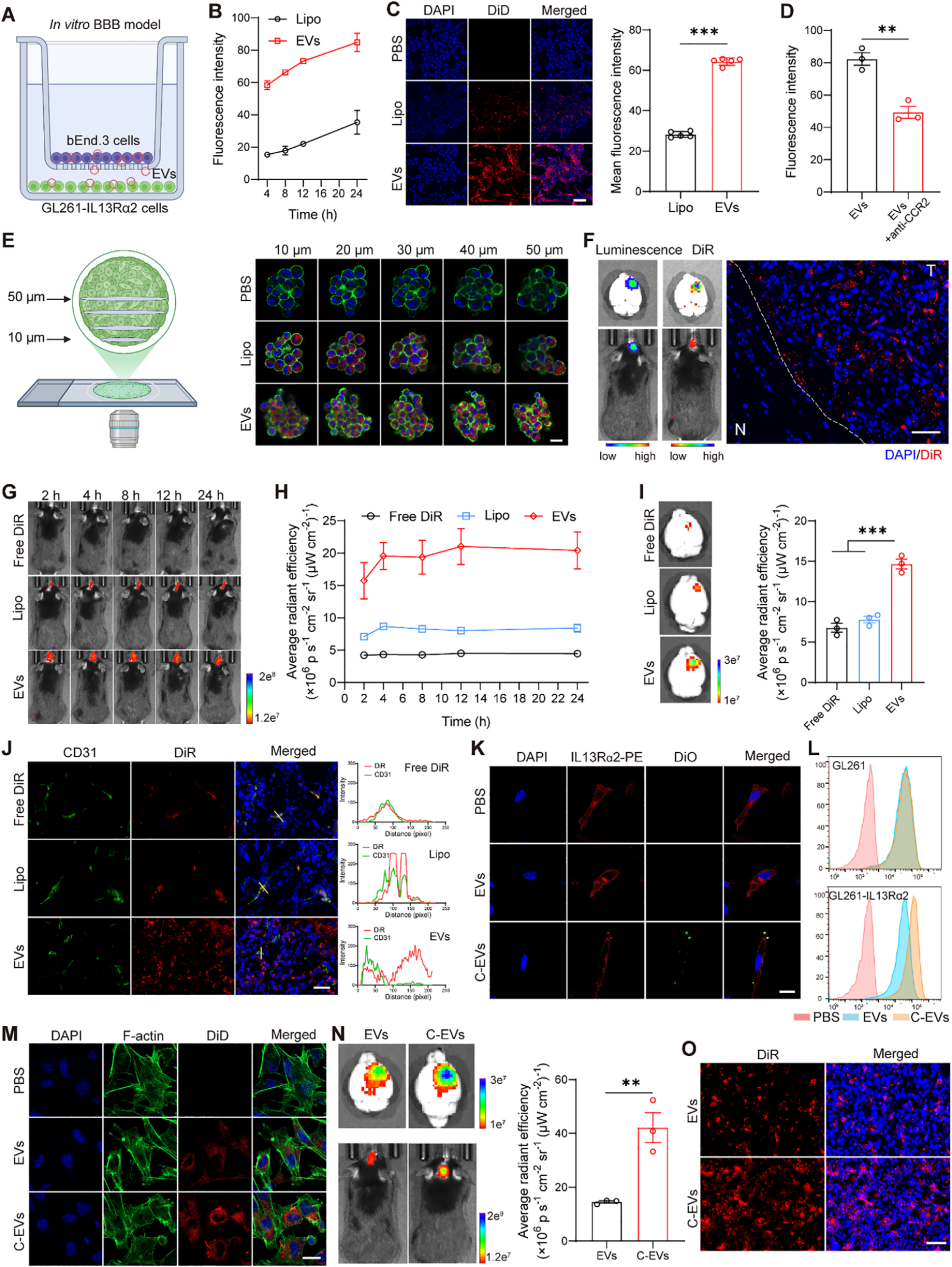
C‐EVs‐mediated enhanced BBB permeability, excellent targeting ability, and brain tumor accumulation. (A) Schematic illustration of in vitro BBB model. (B) Transport ratio of liposome and EVs traverses the BBB model after different time periods (*n* = 3). (C) Confocal images and relative fluorescence intensity of GL261‐IL13Rα2 cells in the lower chamber after treated with DiD‐labeled liposome or EVs for 24 h (*n* = 5). Scale bar, 10 µm. (D) Flow cytometry analysis of fluorescence intensity of GL261‐IL13Rα2 in the lower chamber after incubated with EVs or EVs with anti‐CCR2 (*n* = 3). (E) Schematic illustration of the 3D tumor spheroids and penetration of DiD‐labeled liposome or EVs into GL261‐IL13Rα2 tumor spheroids after 4 h incubation. Scale bar, 50 µm. (F) In vivo and ex vivo bioluminescence and fluorescence imaging of GL261‐IL13Rα2 tumor‐bearing mice and brain at 24 h after tail vein injection of DiR‐labeled EVs. Immunofluorescence staining of tumor‐bearing brain, dotted lines demarcate the tumor boundary (T), with adjacent normal brain tissue (N) shown for anatomical reference. Scale bars: 50 µm. (G and H) Representative fluorescence images (G) and their quantitative analysis (H) of GL261‐IL13Rα2‐bearing mice after *i.v*. injection with free DiR, DiR‐labeled liposome, or EVs at different time points. (I) Ex vivo images of the GL261‐IL13Rα2 bearing brain and their quantification of the fluorescence signal in the brain (*n* = 3). (J) Immunofluorescence staining and the corresponding line profiles of the tumor‐bearing brain after tail vein injection of free DiR, DiR‐labeled liposome, or EVs. DAPI (blue) stained nuclei, and CD31 (green) labeled blood vessels. Scale bar, 50 µm. (K) C‐EVs bound to the membrane of GL261‐IL13Rα2 cells. Scale bar, 10 µm. (L) Degree of cellular uptake of EVs and C‐EVs in GL261 and GL261‐IL13Rα2 quantified by flow cytometry. (M) CLSM images of GL261‐IL13Rα2 cells incubated with EVs and C‐EVs at 4 h. Scale bar, 10 µm. (N) In vivo and ex vivo fluorescence imaging, and their quantification of the fluorescence signal of GL261‐IL13Rα2 tumor‐bearing mice and brain at 24 h after tail vein injection of DiR‐labeled EVs or C‐EVs. (O) Immunofluorescence staining of tumor‐bearing brain after *i.v*. injection with DiR‐labeled EVs or C‐EVs. Scale bars: 100 µm. Statistical analysis was performed by unpaired two‐tailed *t*‐test (C,D and N) or one‐way ANOVA with Tukey's multiple comparisons tests (I). The experimental data were presented as mean ± S.E.M. (*n* = 3). ^*^ *p* < 0.05, ^**^ *p* < 0.01, ^***^ *p* < 0.001.

To better specifically target IL13Rα2‐overexpressing glioma (Figure S9A,B), C‐EVs with glioma antigen‐specific CAR against IL13Rα2 were designed. In vitro validation showed C‐EVs exhibited greater binding to GL261‐IL13Rα2 cells compared to non‐targeting EVs (Figure 3K). Notably, flow cytometric analysis showed that there was no significant difference in binding to GL261 cells between EVs and C‐EVs, indicating that the non‐specific interactions of C‐EVs with IL13Rα2‐negative cells was negligible (Figure 3L). This target specificity was further corroborated by confocal microscopy, which clearly showed preferential accumulation and higher uptake efficiency of C‐EVs on GL261‐IL13Rα2 cells (Figure 3M). Extending these findings to an in vivo model, a 2‐fold increase in tumor accumulation of C‐EVs relative to non‐targeting EVs was observed (Figure 3N), a result that was independently validated by immunofluorescence analysis of brain sections (Figure 3O). The ex vivo organ distribution data (Figure S9C,D) show that, apart from the brain, EVs were distributed mainly in the liver and spleen, consistent with the typical clearance pathways of systemically administered EVs. These results demonstrated that CAR confers precise targeting capabilities to EVs while maintaining their inherent tumor‐homing properties, providing a promising strategy for glioma‐specific drug delivery.

### 2.4 Dual‐Targeting Strategy Enhances Anti‐Tumor Phagocytosis

The CD47‐SIRPα axis is a critical immune checkpoint that allows tumor cells to evade phagocytosis by macrophages [48]. Comparative analysis revealed significantly elevated CD47 expression in glioma tissues versus healthy brain (Figure S9E,F), establishing this pathway as a key therapeutic target. To disrupt this axis, EVs derived from macrophages overexpressing a SIRPα variant (S‐EVs) were engineered. This S‐EVs competitively binds CD47 on tumor cells, preventing endogenous SIRPα‐mediated inhibition of phagocytosis. In vitro phagocytosis assays demonstrated that S‐EVs significantly enhanced the phagocytosis of GL261‐IL13Rα2 glioma cells by RAW 264.7 macrophages (Figure 4A). The efficacy was both dose‐dependent (Figure 4B) and time‐dependent (Figure 4C), confirming that S‐EVs require sustained exposure to maximize phagocytic activity. Despite CD47 expression on red blood cells (RBCs), S‐EVs did not induce detectable hemagglutination (Figure S9G), and flow cytometry showed an increase in RBC binding with an overall rate remaining low, confirming their favorable hemocompatibility (Figure S9H).

**FIGURE 4:**
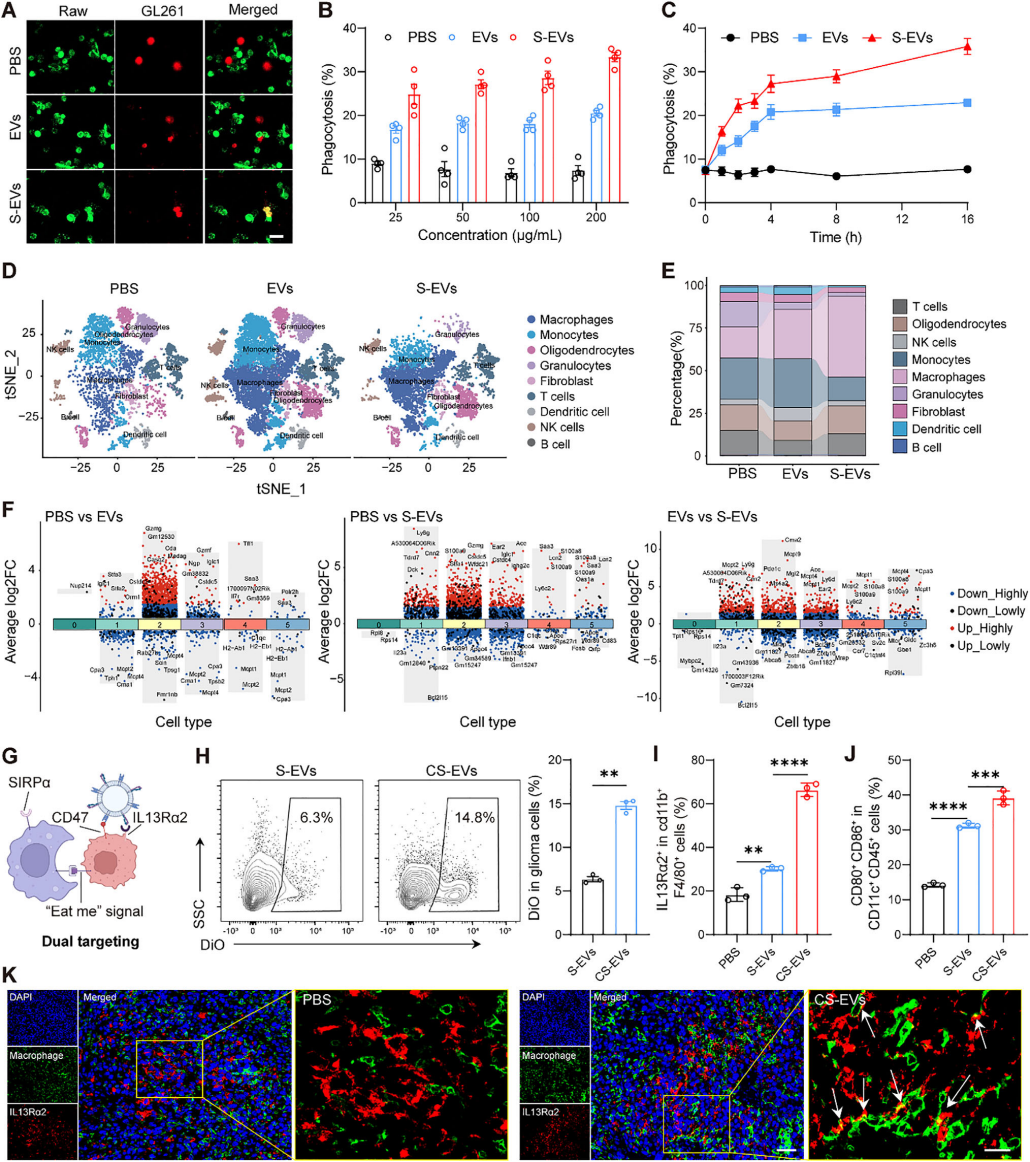
S‐EVs boost anti‐tumor immunity by blocking CD47‐SIRPα, and dual‐targeting strategy further amplifies macrophage phagocytosis. (A) Fluorescence images of phagocytosis assays and quantification analysis of the phagocytosis of GL261‐IL13Rα2 cells by RAW 264.7 cells. Scale bar, 20 µm. (B,C) Quantification analysis of the phagocytosis of GL261‐IL13Rα2 cells by RAW 264.7 cells with different concentrations (B) or different incubation time periods (C) (*n* = 4). (D) t‐SNE map of CD45^+^ cell clusters from tumor‐bearing brain after different treatments. (E) Relative proportions of different types of immune cells in CD45^+^ cells. (F) Gene differential expression compared between PBS, EVs, and S‐EVs NPs treatment groups. 0–5 represent B cells, granulocytes, macrophages, monocytes, NK cells, T‐cells. (G) Schematic illustration of the dual‐targeting strategy combining glioma‐specific antigen recognition with localized CD47‐SIRPα checkpoint blockade. (H) Flow cytometry analysis of DiO‐labeled S‐EVs and CS‐EVs targeting in glioma tissues (*n* = 3). (I) Flow cytometric analysis of IL13Rα2^+^ in tumor tissues, gating on F4/80^+^CD11b^+^CD45^+^ cells after treated with PBS, S‐EVs, or CS‐EVs (*n* = 3). (J) Flow cytometric analysis of CD80^+^CD86^+^ DCs in the tumor tissues gating on CD45^+^CD11c^+^ cells after the indicated treatments (*n* = 3). (K) Immunofluorescence analysis of IL13Rα2^+^ glioma cells and macrophages in GL261‐IL13Rα2 tumor‐bearing mice following treatment with PBS or CS‐EVs. Scale bar, 50 µm. Scale bar of enlarged images, 20 µm. Statistical analysis was performed by unpaired two‐tailed *t*‐test (H) or one‐way ANOVA with Tukey's multiple comparisons tests (I,J). The experimental data were presented as mean ± S.E.M. ^*^ *p* < 0.05, ^**^ *p* < 0.01, ^***^ *p* < 0.001, ^****^ *p* < 0.0001.

To elucidate the systemic immunomodulatory effects of S‐EVs, single‐cell RNA sequencing (scRNA‐seq) was performed on immune cells isolated from GL261‐IL13Rα2 gliomas. t‐SNE clustering (Figure 4D) resolved 9 distinct immune cell populations, including macrophages, monocytes, oligodendrocytes, granulocytes, fibroblast, T cells, DCs, NK cells, and B cells, revealing that S‐EVs treatment significantly altered myeloid composition. Proportional analysis demonstrated S‐EVs‐driven remodeling of the tumor immune landscape, characterized by a decrease in monocytes and an increase in macrophages (Figure 4E). Crucially, differential gene expression analysis (Figure S9I–K) showed that S‐EVs upregulated antigen presentation genes (CD83) and while downregulating immunosuppressive myeloid‐derived suppressor cell (MDSC)‐associated markers (S100A8 and S100A9). To dissect the transcriptional impact of S‐EVs on glioma‐associated immune cells, comparative gene expression analysis was performed. Heatmap analysis revealed that S‐EVs treatment induced a striking upregulation of antigen presentation‐related genes in macrophages and DCs (Figure 4F). Conversely, immunosuppressive markers associated with MDSCs were significantly downregulated in monocytes, validating the S‐EVs‐driven shift from immunosuppressive to immunostimulatory states. These data mechanistically link CD47‐SIRPα blockade to a remodeled myeloid landscape, where enhanced phagocytosis and antigen presentation synergize to activate adaptive immunity, providing a transcriptional blueprint for anti‐tumor efficacy of S‐EVs.

Building on the demonstrated capacity of C‐EVs to enhance antigen recognition and S‐EVs to block the CD47‐SIRPα checkpoint, a dual‐targeting CS‐EV platform that synergistically combines tumor antigen specificity with immune checkpoint blockade to potentiate macrophage phagocytosis was engineered (Figure 4G). In orthotopic GL261‐IL13Rα2 glioma‐bearing mice, dual‐targeting CS‐EVs demonstrated significantly enhanced uptake by glioma cells relative to single‐target S‐EVs (Figure 4H), validating the synergistic targeting advantage of antigen recognition with CD47‐SIRPα blockade. The dual‐targeting CS‐EVs markedly increased macrophage‐mediated engulfment of IL13Rα2^+^ glioma cells (Figure 4I), and immunofluorescence imaging vividly captured the active phagocytosis of tumor cells by macrophages (Figure 4K). This enhanced tumor clearance generated elevated tumor antigen availability, driving DC maturation as evidenced by upregulated CD80/86 expression (Figure 4J), which in turn primed CD8^+^ T cell responses, thereby amplifying systemic anti‐tumor immunity (Figure S9L). Collectively, these results demonstrate that the dual‐targeting CS‐EV platform addresses critical shortcomings of single‐target therapies by spatially and temporally coupling CD47‐SIRPα blockade with antigen‐specific recognition, ultimately achieving superior phagocytic clearance and antigen‐specific adaptive immune responses against glioma.

### 2.5 M1‐CS‐EVs Exhibit Potent Anti‐Tumor Effects Against GL261‐IL13Rα2 Tumor‐Bearing Mice

To assess the therapeutic potential of M1‐CS‐EVs, orthotopic GL261‐IL13Rα2 glioma‐bearing mice were established (Figure 5A). Bioluminescence imaging showed that M1‐CS‐EVs treatment induced a 90% reduction in tumor signal intensity by day 24 compared to the PBS group (Figure 5B,C), accompanied by a significant survival benefit, with a survival of 58 days compared to 27 days in the PBS group (Figure 5D). Multiplex cytokine analysis demonstrated that M1‐CS‐EVs treatment elevated pro‐inflammatory mediators TNF‐α, IFN‐γ, and IL‐6 while suppressing immunosuppressive cytokines IL‐10 (Figure 5E). Histopathological evaluation of M1‐CS‐EVs‐treated tumors further confirmed minimal residual tumor burden (Figure 5F), markedly enhanced apoptosis with TUNEL positive cells (Figure 5G), and significantly reduced proliferation evidenced by decreased Ki‐67 positive cells (Figure 5H). Notably, comprehensive toxicity assessment revealed no evidence of organ damage or hematological abnormalities (Figure S10A).

**FIGURE 5:**
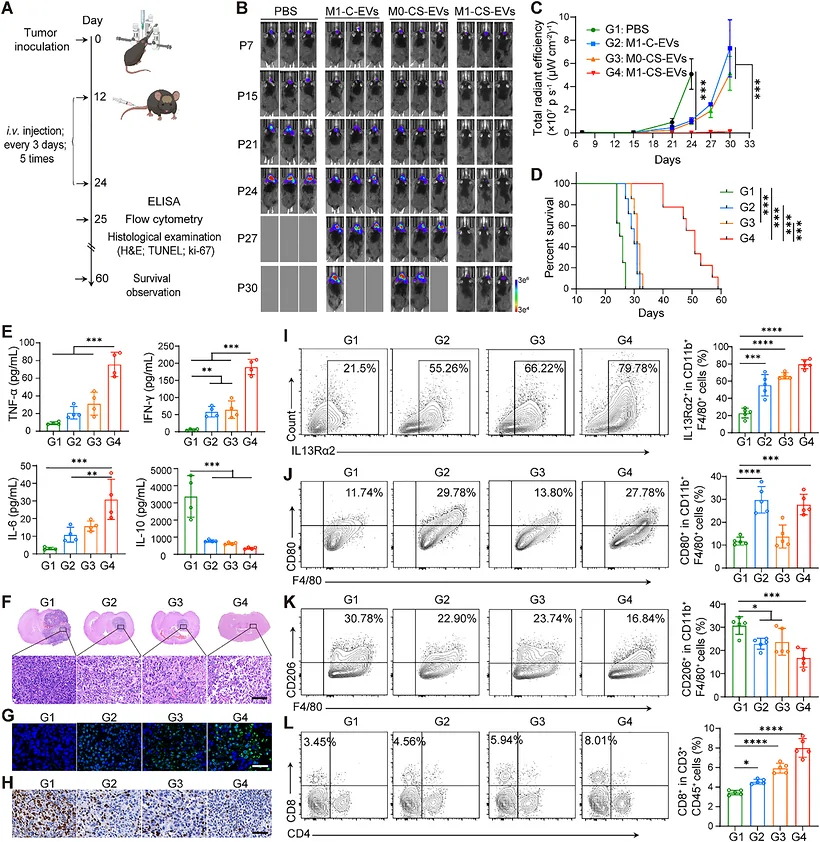
In vivo evaluation of the anti‐tumor effects of M1‐CS‐EVs in GL261‐IL13Rα2‐luc tumor‐bearing mice. (A) Experimental design for evaluating the efficiency of tumor inhibition upon treatments with PBS, M1‐C‐ EVs, M0‐CS‐EVs, and M1‐CS‐EVs in GL261‐IL13Rα2‐luc tumor‐bearing model mice. (B) Representative bioluminescence images showing GL261‐IL13Rα2‐luc tumor progression in mice following intravenous treatment with different experimental groups at specified time points. Blank areas indicate time points where mice succumbed to tumor burden. (C) Quantitative analysis of total bioluminescent flux derived from panel (B) (*n* = 6). (D) Survival curves comparing treatment groups in orthotopic glioma‐bearing mice (*n* = 6). (E) Cytokine profiling (TNF‐α, IFN‐γ, IL‐6, and IL‐10) in tumor tissues post‐treatment, measured by ELISA (*n* = 4). (F) H&E staining of GL261‐IL13Rα2 tumor‐bearing mice in each group after the indicated treatments. Scale bar, 50 µm. (G,H) Immunohistochemical staining of the tumor Ki‐67 (G) and immunofluorescence analysis of TUNEL (H) of GL261‐IL13Rα2 tumor‐bearing mice in each group after the indicated treatments. Scale bar, 50 µm. (I–L) Flow cytometric analysis of IL13Rα2^+^ in tumor tissues gating on F4/80^+^CD11b^+^CD45^+^ cells (I), anti‐tumor macrophages (CD80^+^) (J) and pro‐tumor macrophages (CD206^+^) (K) in tumor tissues gating on F4/80^+^CD11b^+^CD45^+^ cells, and CD8^+^ T cells in the tumor tissues from GL261‐IL13Rα2 tumor‐bearing mice gating on CD3^+^CD45^+^ cells (L) after the indicated treatments (*n* = 5). Statistical analysis was performed by two‐way ANOVA with a Tukey's test (C), log‐rank (Mantel‐Cox) test (D) or one‐way ANOVA with Tukey's multiple comparisons tests (E and I to L). The experimental data were presented as mean ± S.E.M. ^*^ *p* < 0.05, ^**^ *p* < 0.01, ^***^ *p* < 0.001, ^****^ *p* < 0.0001.

Tumor‐infiltrating immune cells were analyzed to further elucidate the mechanisms underlying the anti‐tumor effects of M1‐CS‐EVs (Figure S11). Flow cytometry analysis revealed a marked increase in IL13Rα2^+^ tumor cells engulfed by macrophages after M1‐CS‐EVs treatment (Figure 5I). Furthermore, a significant increase in anti‐tumor macrophages (Figure 5J) and CD8^+^ T cells (Figure 5L), along with a decrease in pro‐tumor macrophages (Figure 5K) was observed in the M1‐CS‐EVs‐treated tumors. Immunofluorescence staining of tumor sections confirmed enhanced infiltration of anti‐tumor macrophages and CD8^+^ T cells, along with reduced pro‐tumor macrophages (Figure S10B–D). The significant changes in immune cell populations highlight the dual role of M1‐CS‐EVs in both directly targeting tumor cells and modulating the immune landscape to achieve a robust anti‐tumor response.

To validate the broad therapeutic potential of our engineered M1‐CS‐EVs, their efficacy was evaluated in T cell‐deficient nude mice bearing human U87 glioma, which express IL13Rα2 (Figure S12A). Despite the immunocompromised status, M1‐CS‐EVs maintained their tumor‐targeting capability, showing significantly higher cellular uptake than untargeted EVs (Figure S12B,C) and enhanced tumor accumulation in vivo (Figure S12D–I). Similar to the GL261 model, M1‐CS‐EVs treatment led to a significant reduction in tumor growth, as evidenced by bioluminescence imaging (Figure S13A–C). Flow cytometry analysis of tumor‐infiltrating immune cells showed an enhanced phagocytic activity of macrophages toward tumor cells, an increase in anti‐tumor macrophages and a decrease in pro‐tumor macrophages, consistent with the findings in the GL261 model (Figure S13D–F). Although the therapeutic effect was moderately attenuated compared to immunocompetent hosts, these findings confirm that M1‐CS‐EVs can effectively target IL13Rα2‐positive tumors and remodel the innate immune microenvironment independent of adaptive immunity, supporting their potential as a versatile platform for diverse glioma subtypes.

### 2.6 In Vivo Evaluation of the Anti‐Tumor Effects of M1‐CS‐EVs in CT‐2A‐EGFRvIII Tumor‐Bearing Mice

Building on our previous demonstration that M1‐CS‐EVs exhibited potent anti‐tumor activity in GL261‐IL13Rα2 glioma models, we further engineered our platform to target EGFRvIII (Figure S14A), a tumor‐specific antigen frequently overexpressed in gliomas [6]. To validate the versatility of this platform, M1‐CS‐EVs were reconfigured by replacing the IL13Rα2‐targeting domain with an EGFRvIII‐targeting domain. Dual‐target‐expressing macrophages were successfully prepared (Figure 6A,B), and their derived CS‐EVs were isolated (Figure 6C). EVs and C‐EVs were co‐incubated with CT‐2A‐EGFRvIII cells, and confocal microscopy revealed enhanced binding of C‐EVs to CT‐2A‐EGFRvIII cells compared with EVs (Figure 6D). Meanwhile, tumor‐targeting domain modification significantly increased cellular uptake in CT‐2A‐EGFRvIII cells, confirming antigen‐specific targeting (Figure S14B–D). The fluorescence intensity of C‐EVs at the tumor site was significantly higher than that of EVs (Figure 6E,F). Immunofluorescence staining of CT‐2A‐EGFRvIII tumor‐bearing brain sections confirmed that the in vivo tumor accumulation of C‐EVs in EGFRvIII‐overexpressing gliomas was higher than that of EVs (Figure S14E), indicating that CAR effectively increased the accumulation within the tumor.

**FIGURE 6:**
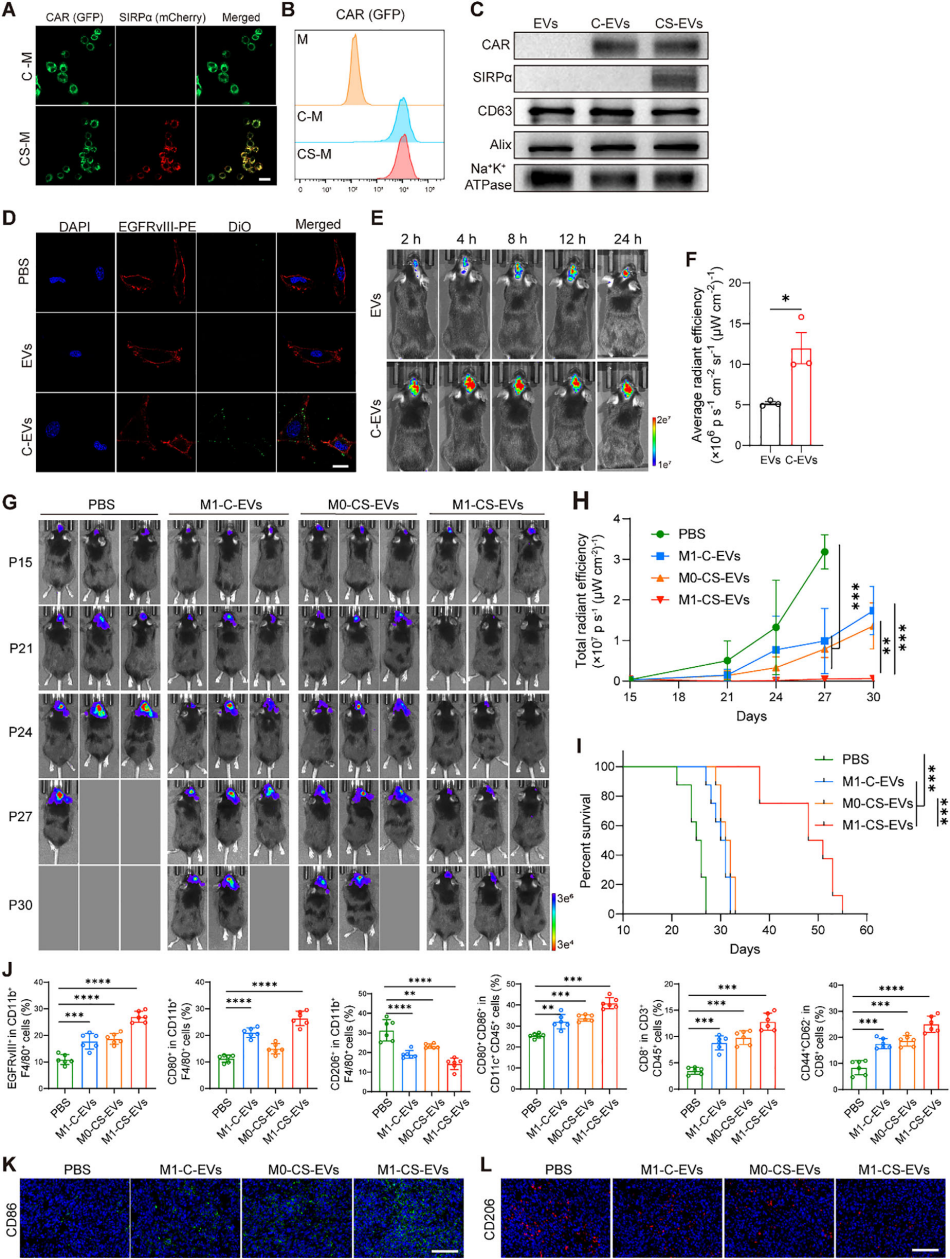
In vivo evaluation of the anti‐tumor effects of M1‐CS‐EVs in CT‐2A‐EGFRvIII‐luc tumor‐bearing mice. (A) Immunofluorescence imaging of CAR (GFP) and SIRPα (mCherry) expression on C‐M and CS‐M. Scale bar, 5 µm. (B) Flow cytometry analysis of CAR (GFP) and SIRPα (mCherry) expression on M, C‐M, and CS‐M. (C) Western blotting analysis of CAR, SIRPα, CD63, Alix, and Na^+^K^+^ ATPase in various EVs. (D) C‐EVs bound to the membrane of CT‐2A‐EGFRvIII cells. Scale bar, 10 µm. (E) In vivo fluorescence imaging of CT‐2A‐EGFRvIII tumor‐bearing mice with i.v. injection of EVs and C‐EVs at different time points. (F) Quantification of ex vivo fluorescence signal in the CT‐2A‐EGFRvIII‐bearing brain after *i.v*. injection with EVs and C‐EVs (*n* = 3). (G) Representative bioluminescence images showing CT‐2A‐EGFRvIII‐luc tumor progression in mice following intravenous treatment with different experimental groups at specified time points. Blank areas indicate time points where mice succumbed to tumor burden. (H) Quantitative analysis of total bioluminescent flux derived from panel (g) (*n* = 6). (I) Survival curves comparing treatment groups in orthotopic glioma‐bearing mice (*n* = 6). (J) Flow cytometric analysis of EGFRvIII^+^ in tumor tissues gating on F4/80^+^CD11b^+^CD45^+^ cells, anti‐tumor macrophages (CD80^+^) and pro‐tumor macrophages (CD206^+^) in tumor tissues gating on F4/80^+^CD11b^+^CD45^+^ cells, CD80^+^CD86^+^ DCs in the tumor tissues gating on CD45^+^CD11c^+^ cells, CD8^+^ T cells in the tumor tissues gating on CD3^+^CD45^+^ cells, and CD44^+^CD62L^−^ T cells in the tumor tissues gating on CD8^+^ cells after the indicated treatments (*n* = 5). (K,L) Immunofluorescence analysis of CD86 (K) and CD206 (L) of CT‐2A‐EGFRvIII tumor‐bearing mice in each group at the end of therapy. Scale bar, 50 µm. Statistical analysis was performed by unpaired two‐tailed *t*‐test (F), two‐way ANOVA with Tukey's test (H), log‐rank (Mantel‐Cox) test (I) or one‐way ANOVA with Tukey's multiple comparisons tests (J). The experimental data were presented as mean ± S.E.M.^*^ *p* < 0.05, ^**^ *p* < 0.01, ^***^ *p* < 0.001, ^****^ *p* < 0.0001.

In CT‐2A‐EGFRvIII orthotopic glioma‐bearing mice, systemic administration of M1‐CS‐EVs significantly suppressed tumor progression (Figure 6G). Tumor volume reduction and prolonged survival were achieved without observable off‐tumor toxicity (Figure 6H,I). Importantly, the anti‐tumor efficacy of EGFRvIII‐targeted‐EVs mirrored the potency observed in IL13Rα2‐targeted EVs, confirming that our modular targeting system retains functional consistency across distinct antigen targets. Flow cytometry revealed that M1‐CS‐EVs uniquely remodeled the tumor immune landscape, increasing anti‐tumor macrophages, activated DCs, and tumor‐infiltrating CD8, while suppressing pro‐tumor macrophages (Figure 6J). Immunofluorescence confirmed enhanced CD86 and diminished CD206 in the M1‐CS‐EVs group (Figure 6K,L). These results demonstrate that the synergistic integration of monocyte differentiation into anti‐tumor macrophages and dual‐targeting strategy driving localized CD47‐SIRPα blockade achieves superior anti‐tumor immunotherapeutic effects by reversing immunosuppression and activating anti‐tumor immune responses.

## 3 Discussion

In this study, we developed a promising immunotherapeutic strategy by exploiting M1‐EVs to induce monocyte differentiate into anti‐tumor macrophage. Through single‐cell transcriptomics, we identified TNF‐α signaling as the key mechanism by which M1‐EVs drive anti‐tumor macrophage differentiation. To further enhance the anti‐tumor activity, we used engineered EVs to co‐express a tumor‐targeting CAR against IL13Rα2 or EGFRvIII and an immune checkpoint‐targeting module featuring SIRPα variants to block the CD47‐SIRPα pathway. These M1‐CS‐EVs demonstrated excellent BBB penetration, glioma targeting, and potent anti‐tumor effects in orthotopic glioma models. By disrupting immune evasion and activating the innate immune function of phagocytes, M1‐CS‐EVs effectively suppressed tumor growth and prolonged survival without observable off‐target toxicity.

Our study challenges the traditional approach of depleting or repolarizing tumor‐associated macrophages (TAMs) and instead seeks to induce monocytes to differentiate into anti‐tumor macrophages. The efficacy of the traditional approach remains limited because TAMs have a high turnover rate and are constitutively replenished by circulating monocytes [15]. By redirecting monocyte differentiation through M1‐EVs, a durable anti‐tumor macrophage population was obtained that was able to resist microenvironmental immunosuppression [23]. This approach not only addresses the challenges in glioma therapy, but also utilizes the body's own cell renewal system as a sustained drug delivery platform. More broadly, the work illuminates a path toward precision monocyte engineering, not just for cancer but for any condition where immune cell behavior dictates outcomes.

The EV‐based platform offers unique advantages for glioma therapy, combining natural BBB penetration and immune evasion capabilities, properties that cannot be replicated by synthetic nanoparticles [55]. Building on these inherent advantages, our engineered EV‐based platform introduces modular customization while retaining EV functionality. The modular design of the EV‐based platform allows for rapid customization to target different tumor antigens, addressing the challenge of antigen heterogeneity in glioma. This design maintains optimal EV size for consistent biodistribution, while the modular design has applications beyond oncology. The plug‐and‐play flexibility of this system makes the EV‐based platform a transformative approach to precision medicine for glioma.

## 4 Experimental Section

### 4.1 Cell Lines and Cell Culture

Mouse glioma GL261 cells, mouse glioma CT‐2A cells, human glioblastoma U87MG cells, RAW 264.7 macrophage, and mouse brain endothelial cells (bEnd.3) were purchased from the American Type Culture Collection (ATCC). GL261‐IL13Rα2 and CT‐2A‐EGFRvIII cells were established by infecting GL261 and CT‐2A cells with a lentivirus expressing human IL13Rα2 and EGFRvIII, respectively. The IL13Rα2‐ and EGFRvIII‐positive subpopulations were then sorted by flow cytometry using IL13Rα2‐PE and EGFRvIII‐PE antibodies according to the product instructions. Luciferase‐transfected glioma cell lines were created by transfecting GL261‐IL13Rα2, CT‐2A‐EGFRvIII, and U87MG cells with lentivirus expressing luciferase and puromycin resistance genes. To develop IL13Rα2‐specific CAR macrophages and EGFRvIII‐specific CAR macrophages, RAW 264.7 cells were sorted and subcloned after transfection with lentivirus carrying CAR composed of the human IL13Rα2 or EGFRvIII tumor‐targeting domain (scfv), a short hinge (SSR), and the CD28 transmembrane domain (CD28TM), and then screened with puromycin. SIRPα variant‐overexpressed macrophages were established by infecting RAW 264.7 cells with lentivirus expressing SIRPα variant fused with murine SIRPα transmembrane domain and selecting with puromycin. Macrophages co‐overexpressing CAR and SIRPα were generated by infecting RAW 264.7 cells with lentivirus expressing both CAR and SIRPα variant and selecting with puromycin. All of the cells were cultured in DMEM containing 10% FBS, 1% streptomycin, 1% penicillin, and incubated at 37°C in a humidified incubator with 5% CO_2_.

### 4.2 Animals and Orthotopic Glioma‑Bearing Model

C57BL/6 mice (female, 6–8 weeks) and BALB/c nude mice (female, 6–8 weeks) were purchased from Bestest Biotechnology (Zhuhai, China). All animal experimental protocols were performed in accordance with the regulations of the Institutional Animal Care and Use Committee (IACUC) at the Animal Experiment Center of Shenzhen Bay Laboratory, and all animal experiments were approved by the Animal Ethics Committee of Shenzhen Bay Laboratory (Ethics No. AERL202401).

The orthotopic glioma‐bearing model was established by injecting a luciferase‐labeled GL261‐IL13Rα2 cell suspension into the right striatum of C57BL/6 mice. Briefly, the mice were anesthetized with 0.25% tribromoethanol and then immobilized on a stereotaxic apparatus. The skull was punctured using a drill at the following coordinates: 2 mm lateral to and 1 mm anterior to the bregma. A microsyringe was inserted into the burr hole to a depth of 3 mm below the surface of the dura, and approximately 3 × 10^5^ cells were slowly injected into the cavity. The microsyringe was held at the injection point for 5 min before being slowly withdrawn. Postoperatively, the mice were monitored after scalp suturing until they resumed normal activities. The growth of the intracranial tumor was tracked using bioluminescence imaging. Similarly, orthotopic U87MG and CT‐2A‐EGFRvIII glioma‐bearing models were established by injecting Luciferase‐ labeled U87MG and CT‐2A‐EGFRvIII cell suspension into the right striatum of nude mice and C57BL/6 mice. Bioluminescence imaging was also employed to track the growth of intracranial tumors.

### 4.3 Isolation and Characterization of EVs

Macrophage‐derived EVs was isolated and purified from cell culture supernatants by ultracentrifugation. In brief, cells were cultured for 48 h in EVs‐depleted medium, which was prepared by preprocessing FBS with ultracentrifugation at 200 000 g for 3 h. The cell culture supernatant was sequentially centrifuged at 300 and 2000 g for 10 min, followed by centrifugation at 10 000 g for 30 min to remove cell debris. Subsequently, the supernatant was subjected to ultracentrifugation at 100 000 g for 2 h at 4°C, yielding a pellet enriched with EVs. The obtained EVs were resuspended in PBS and ultracentrifuged again at 100 000 g for 2 h. The final pellet was resuspended in PBS and stored at −80°C until use. To obtain M1‐like CAR macrophage‐derived EVs (M1‐C‐EVs), CAR macrophages were treated with 1 µg/mL lipopolysaccharide (LPS; Merck) to induce an M1‐like phenotype. Subsequently, cell culture supernatants were collected and isolated following the standard protocol described above. Similarly, EVs derived from macrophages co‐overexpressing CAR and SIRPα in a resting state (M0‐CS‐EVs) or an M1‐like state (M1‐CS‐EVs) were obtained using the same protocol. The final EVs was resuspended in PBS and stored at −80°C until use.

The concentration of EVs was determined using a BCA kit. The expression of CAR, SIRPα, CD63, Alix, and Na^+^K^+^ ATPase of EVs was evaluated by Western blotting. For the proteinase K digestion assay, CS‐EVs were incubated with proteinase K at 37°C for 30 min. Samples were then analyzed by Western blotting using antibodies against CAR, SIRPα, and Alix. The protein profile of EVs was analyzed by Sodium dodecyl sulfate‐polyacrylamide gel electrophoresis (SDS‐PAGE). The morphology of EVs was verified using TEM (Talos F200X) and Cryo‐TEM (Thermo Scientific Tundra). The hydrate particle size and zeta potential of EVs were performed using a Zetasizer Nano‐ZS (Malvern Instruments). The size distribution of EVs was detected using an NS 300 (Malvern Instruments). For stability assessment, particle size and zeta potential of EVs in PBS was monitored by DLS at indicated time points during 8 days. For serum stability, EVs were incubated in PBS containing 10% FBS at 37°C for 48 h, and measurements were taken at 0, 4, 12, 24, and 48 h. Moreover, TEM imaging was performed on freshly prepared EVs and on EVs after 8 days of storage to evaluate morphological integrity.

### 4.4 Differentiation of Monocytes Into Anti‐Tumor Macrophages

Monocytes were isolated from bone marrow of C57BL/6 mice. Briefly, the tibiae and femurs were dissected and cut open with scissors. The bone marrow was flushed out with DMEM medium, and the resulting cell suspension was treated with red blood cells (RBCs) lysis buffer to lyse the RBCs. The isolated cells were seeded into 6‐well plates and cultured for one week. Subsequently, the cells were treated with PBS, M0‐EVs, or M1‐EVs for 24 h. The expression of monocyte (Ly‐6C), anti‐tumor macrophage (CD86), and pro‐tumor macrophage (CD206) markers was assessed using flow cytometry. Additionally, the mRNA expression levels of M1 markers (TNF‐α and iNOS) and M2 markers (Mrc1 and Fizz‐1) were analyzed using Quantitative real‐time polymerase chain reaction (qRT‐PCR). To distinguish between TNF‑α protein and mRNA, monocytes were treated with M1‑EVs pre‑incubated with anti‑TNF‑α neutralizing antibody (BioLegend). For RNA degradation, M1‑EVs were treated with RNase A (Thermo Fisher Scientific), followed by washing. After 24 h, Ly‑6C^+^ monocytes among CD11b^+^ cells were analyzed by flow cytometry.

### 4.5 Microglial Polarization Assay

BV‐2 microglial cells were seeded in 12‐well plates and cultured overnight. Cells were then treated with PBS, M0‐EVs, or M1‐EVs for 24 h. After treatment, cells were harvested, washed with PBS, and stained with anti‐CD80‐BV421, and anti‐CD206‐PerCP‐Cy5.5. After washing, the cells were analyzed by flow cytometry.

### 4.6 Phagocytosis of Cancer Cells by Macrophages

GL261‐IL13Rα2 cells were first treated with EVs or S‐EVs. Subsequently, RAW 264.7 cells were stained with CellTracker Green (Thermo Fisher Scientific), while GL261‐IL13Rα2 cells were labeled with CellTracker Red (Thermo Fisher Scientific). The fluorescence‐labeled RAW 264.7 and GL261‐IL13Rα2 cells were then co‐cultured for 4 h. The phagocytosis of GL261‐IL13Rα2 cells by RAW 264.7 cells was quantified by measuring the red fluorescence signal associated with the formation of phagosomes within RAW 264.7 cells.

### 4.7 Transwell Chemotaxis Assay

GL261‐IL13Rα2 cells were seeded in 12‐well plates and treated with PBS, M0‐EVs, or M1‐EVs for 24 h. Subsequently, the media were collected, centrifuged at 300 × g for 5 min, and filtered through 0.45 µm filters to remove cell debris and residual EVs. For the transwell assay, 600 µL of collected media were added to the lower chamber of 24‐well transwell plates (8 µm pore size). Then, 200 µL of monocyte suspension (1 × 10^5^ cells) were added to the upper chamber. After incubation at 37°C for 4 h, cells that migrated to the lower chamber were collected and counted.

### 4.8 In Vitro Cytotoxicity Assay

The cytotoxicity of EVs on macrophages, T cells, DCs, and tumor cells was evaluated using the cell counting kit‐8 (CCK‐8) assay (Beyotime, China). Briefly, RAW264.7, Jurkat, DC2.4, and GL26‐IL13Rα2 cells were seeded in 96‐well plates at a density of 1 × 10^4^ cells per well for 24 h. Subsequently, the cells were treated with EVs at varying concentrations (0–100 µg mL^−1^). Following 24 h of incubation, cytotoxicity was measured using the CCK‐8 assay, and cell viability was normalized to that of untreated control cells. For the hemagglutination assay, 50 µL of 2% RBC suspension was mixed with 50 µL of PBS, EVs, or S‑EVs at final concentrations of 0, 12.5, 25, 50, 75, and 100 µg/mL in U‑bottom 96‑well plates. After incubation at 37°C for 2 h, the plates were photographed to evaluate RBC aggregation. For the RBC‑binding assay, 2% RBC suspension was incubated with DiO‑labeled EVs or S‑EVs at 100 µg/mL at 4°C for 1 h. After washing with PBS, RBC‑associated fluorescence was analyzed by flow cytometry.

### 4.9 In Vitro BBB Penetration

The in vitro BBB model constructed using a Transwell cell culture system was used to evaluate the penetration performance of EVs. Briefly, bEnd.3 cells (2.5 × 10^4^ cells per well) were seeded into the upper chamber of a 24‐well Transwell and cultured for 3 days until a monolayer cell was formed, whereas GL261‐IL13Rα2 (1 × 10^3^ cells per well) cells were cultured onto the bottom chamber. Specifically, DiD‐labeled liposome or EVs was added to the upper chamber. The penetration efficiency was determined by collecting samples from the lower chamber. After 4, 8, 12, and 24 h of incubation, the amount of liposome or EVs crossing the bEnd.3 monolayer was analyzed according to DiD fluorescence via flow cytometry. The cellular uptake of liposome or EVs in GL261‐IL13Rα2 was observed by a ZEISS LSM980 confocal laser scanning microscope (CLSM). Neutralizing antibodies against CCR2 (MedChemExpress, USA) were used in antibody‐blocking experiments. Then, DiD‐labeled EVs were pre‐incubated with anti‐CCR2 antibodies according to the manufacturer's instructions before added to the upper chamber. After 8 h of incubation, samples from the lower chamber were collected to determine the penetration efficiency, and the concentration of EVs in the lower chamber was analyzed according to DiD fluorescence.

### 4.10 EVs Penetration Into Tumor Spheroids

GL261‐IL13Rα2 tumor spheroids were established and cultured under non‐adherent culture conditions in ultralow attachment plates as previously described^43^. Subsequently, the spheroids were transferred to confocal dishes at a density of 2 × 10^4^ cells per well and incubated with DiD‐labeled liposome or EVs for 4 h. Following nuclear staining with DAPI and cytoskeletal staining with phalloidin‐FITC, confocal images were captured in different layers of spheroids at 10 µm intervals from the bottom to the middle using CLSM.

### 4.11 Specific Binding Between C‐EVs and IL13Rα2

To investigate the specific binding of C‐EVs to GL261‐IL13Rα2 cells, GL261‐IL13Ra2 cells were seeded at a density of 2 × 10^4^ cells/well in confocal culture dishes and cultured at 37°C for 24 h. The cells were then incubated with fresh cold culture medium containing DiO‐labeled EVs and C‐EVs for another 2 h. The cell nuclei were stained with DAPI, IL13Rα2 was stained with anti‐IL13Rα2‐PE (biolegend), and the cells were observed under an LSM980 confocal microscope.

### 4.12 Cellular Uptake Assay

GL261 or GL261‐IL13Ra2 cells were seeded in a confocal dish for 24 h and then incubated with DiO‐labeled EVs and C‐EVs for another 4 h. Following staining with DAPI (nuclear) and phalloidin‐FITC (F‐actin), cells were observed under CLSM. To assess cellular uptake efficiency, GL261 and GL261‐IL13Rα2 cells were first cultured overnight, then incubated with DiO‐labeled EVs or C‐EVs for 4 h before flow cytometric analysis. U87MG, CT‐2A, and CT‐2A‐EGFRvIII cells were seeded and incubated with DiO‐labeled EVs or C‐EVs as described above, and cellular uptake of EVs were determined by CLSM and flow cytometry.

### 4.13 In Vivo Safety Evaluation

To evaluate potential acute systemic inflammation, mice were injected with PBS or M1‐EVs, and blood samples were collected at 4 h post‐injection. Serum levels of TNF‐α and IL‐6 were measured by ELISA. To assess the in vivo biocompatibility of the EVs, C57BL/6 mice were administered 100 µL of PBS, EVs, M1‐C‐EVs, M0‐CS‐EVs, or M1‐CS‐EVs at 20 mg kg^−1^ through tail vein injection. Four weeks post‐injection, murine blood samples were collected for comprehensive hematological and biochemical profiling, while major organs were harvested for histopathological assessment using hematoxylin and eosin (H&E) staining to evaluate systemic toxicity across treatment groups.

### 4.14 In Vivo Bio‐Distribution

100 µL of PBS, free DiR, DiR‐labeled liposome, or EVs (at a dose of 10 mg kg^−1^) were intravenously injected into GL261‐IL13Rα2 tumor‐bearing mice. Following the injection, the mice were monitored at different time points using the IVIS Spectrum imaging system (IVIS Lumina III, PerkinElmer). Subsequently, mice were sacrificed, and major organs, as well as the tumor‐bearing brains, were harvested for ex vivo tissue imaging.

### 4.15 BBB and Tumor Penetration Assessment

To evaluate BBB and tumor permeability, GL261‐IL13Rα2 tumor‐bearing mice were intravenously injected with PBS, free DID, DiD‐labeled liposome, EVs, or C‐EVs. Tumor‐bearing brains were harvested 24 h post‐injection, and processed for histological analysis through sequential fixation (4% formalin, overnight), paraffin embedding, and sectioning. Immunofluorescent staining was performed on sections, with high‐resolution digital imaging acquired using a Leica Aperio VERSA 200 whole‐slide scanner for quantitative analysis.

### 4.16 In Vivo Anti‐Tumor Study

The therapeutic efficacy of EVs was assessed in mice bearing orthotopic GL261‐IL13Rα2‐luc, U87MG‐luc, or CT‐2A‐EGFRvIII‐luc tumors. The mice were randomly allocated into four groups (ten mice per group, six for survival monitoring and four for histological analysis) and treated with PBS, M1‐C‐EVs, M0‐CS‐EVs, or M1‐CS‐EVs (10 mg kg^−1^) via tail vein injection every 3 days for a total of five doses. The bioluminescence intensity of the glioma for each mouse was monitored and recorded every 3 days. At the experimental endpoint, the tumor‐bearing brains were collected and stained with H&E and Ki‐67, main organs were harvested for histological analysis using H&E staining. Additionally, the tumor‐bearing brains were subjected to TUNEL. To characterize immune cell infiltration, tumor histologic sections were immunofluorescence stained for T cells (CD8), anti‐tumor macrophage (anti‐CD86), and pro‐tumor macrophage (anti‐CD206) and evaluated with a Digital Pathology Scanner. The staining procedures was performed by Servicebio in accordance with the manufacturer's protocols.

### 4.17 Single‐Cell RNA Sequencing of Tumor Tissue

Tumor tissues were processed into a single‐cell suspension after enzymatic digestion, and CD45‐positive cells were sorted by flow cytometry. Droplet‐partioning barcode was performed using the Chromium Single Cell Controller (10x Genomics) system in the NCI‐CCR Single Cell Analysis Tool based on the complementary DNA database of oligo deoxythymidylic acid (oligo dT). Cells were adjusted to the desired concentration, and approximately 10 000 cells were detected on the machine. Sequencing was performed using Illumina NovaSeq at the NCI‐CCR Sequencing Center. Data analysis was performed using the R package Seurat.

### 4.18 Flow Cytometry

Following treatment, tumor tissues were harvested, enzymatically dissociated in supplemented culture medium, and mechanically filtered through 70 µm cell strainers to generate single‐cell suspensions. For subsequent macrophage polarization analysis, the isolated cells were stained with anti‐CD45‐APC‐Cy7, anti‐F4/80‐PE‐Cy7, anti‐CD11b‐FITC, anti‐Ly‐6C‐APC, anti‐CD80‐Bv421, anti‐CD206‐PerCP‐Cy5.5, and anti‐IL13Rα2‐PE for 30 min. For intracellular detection of IL13Rα2, cells were fixed and permeabilized prior to incubation with anti‐IL13Rα2‐PE [64, 65]. For T cells detection, the cells were stained with anti‐CD45‐APC‐Cy7, anti‐CD3‐BUV395, anti‐CD4‐BV650, anti‐CD8‐BV605, anti‐CD62L‐PE‐Cy7, and anti‐CD44‐BB700 for 30 min. To verify the maturation of DC, the cells were stained with anti‐CD45‐APC‐Cy7, anti‐CD11c‐FITC, anti‐CD80‐Bv421, and anti‐CD86‐PE (all from BioLegend) for 30 min. After washing with PBS, the samples were analyzed by flow cytometry.

### 4.19 Cytokine Detection

Tumor‐bearing brains were collected at the end of treatment, and tumor tissues from each group were isolated. The intratumor levels of tumor necrosis factor alpha (TNF‐α), transforming growth factor‐β (TGF‐β), interferon gamma (IFN‐γ), interleukin 6 (IL‐6), IL‐2, IL‐10, and IL‐12 were measured by using corresponding ELISA kits, following the manufacturers' protocols.

### 4.20 Statistical Analysis

All data are presented as mean ± standard error of the mean (S.E.M.) based on independent experiments. Statistical analyses included unpaired two‐tailed *t*‐tests for two‐group comparisons, one‐way or two‐way ANOVA with Tukey's/Dunnett's post hoc tests for multiple group comparisons, and log‐rank (Mantel‐Cox) tests for survival analysis. Statistical significance was defined as *p* < 0.05, significance levels are denoted as follows: (^*^) for *p* < 0.05, (^**^) for *p* < 0.01, (^***^) for *p* < 0.001, and (^****^) for *p* < 0.0001.

## Author Contributions

Y.P., X.L., Q.‐F.M., C.Y., and L.R.: Conceptualization. Y.P., X.L., and Q.‐F.M.: Methodology. Y.P., X.L., Q.‐F.M., L.C., J.Z., and C.Z.: Investigation. Y.P., Q.‐F.M., C.Y., and L.R.: Resources. Y.P., X.L., Q.‐F.M., and L.C.: Visualization. C.Y., and L.R.: Supervision. Y.P., X.L., Q.‐F.M., R.L., and Y.X.: Validation. Y.P., X.L., and Q.‐F.M.: Writing – original draft. C.Y., and L.R.: Writing – review and editing.

## Conflicts of Interest

The authors declare no conflicts of interest.

## Supporting information




**Supporting File**: advs76910‐sup‐0001‐SuppMat.docx.

## Data Availability

The data that support the findings of this study are available from the corresponding author upon reasonable request.
