# Supplementary material for: Redirecting Monocyte Differentiation With Engineered Extracellular Vesicles for Glioma Immunotherapy
Source: Adv Sci (Weinh). 2026 Aug 5;13(59):e76910. doi: 10.1002/advs.76910 (PMC13440210; doi:10.1002/advs.76910)
Supplement: Supplementary file 1 [file ADVS-13-e76910-s001.docx]

*Supporting Information*

**Redirecting Monocyte Differentiation with Engineered Extracellular Vesicles for Glioma Immunotherapy**

Yuanwei Pan^1,3^, Xuan Liu^2,3^, Qian-Fang Meng^1,3^, Lei Cao^1^, Rongrong Li^1^, Yangtao Xu^1^, Jing Zhang^1^, Chenchen Zhao^1^, Lang Rao^1,^*, and Chen Yu^2,^*

^1^ Institute of Chemical Biology, Shenzhen Bay Laboratory, Shenzhen 518132, China.

^2^ Institute of Cancer Research, Shenzhen Bay Laboratory, Shenzhen 518132, China.

^3^ These authors contributed equally.

* Corresponding author: [lrao@szbl.ac.cn](mailto:lang.rao@szbl.ac.cn) (L.R.), and [yu@szbl.ac.cn](mailto:yu@szbl.ac.cn) (Y.C.).

**Supplemental Figures**

**Figure S1. Assessment of the immunomodulatory functions of M1-EVs.**

(A) Relative mRNA expression of TNF-α, INOS, IL-6, and CD86 in M0-EVs and M1-EVs (*n* = 4). (B-D) Flow cytometric analysis of monocyte (Ly-6C^+^) (B), anti-tumor macrophages (CD86^+^) (C) and pro-tumor macrophages (CD206^+^) (D) after monocytes treated with PBS, M0-EVs, or M1-EVs (*n* = 3). (E) Flow cytometric analysis of Ly‑6C⁺ monocytes after treatment with PBS, M1‑EVs, M1‑EVs pre‑incubated with anti‑TNF‑α neutralizing antibody, or M1‑EVs treated with RNase A (*n* = 3). (F) Relative expressions of mRNA in anti-tumor markers (IL-6) and pro-tumor markers (Mrc1, Fizz1, and IL-10) as evaluated by qPCR after treated with PBS, M0-EVs, or M1-EVs (*n* = 4). (G-H) Flow cytometric analysis of anti-tumor macrophages (G) and pro-tumor macrophages (H) in tumor tissues gating on CD11b^+^CD45^+^ cells after the indicated treatments (*n* = 3). (I) TGF-β and (J) IL-12 levels in tumor tissues collected from mice after different treatments (*n* = 4). (K) Representative flow cytometry plots and quantification of CD80⁺ and CD206⁺ cells among BV-2 cells after treatment with PBS, M0-EVs, or M1-EVs (*n* = 3). (L) Quantification of migrated monocytes after incubation with media from glioma cells treated with PBS, M0-EVs, or M1-EVs (*n* = 3). Statistical analysis was performed by unpaired two-tailed t test (A) or one-way ANOVA with Tukey’s multiple comparisons tests (B-L). Data are presented as mean ± S.E.M. **P* < 0.05, ***P* < 0.01, ****P* < 0.001, *****P* < 0.0001.

**Figure S2. Basic information of cell subsets among different groups.**

(A) Heat map of marker gene expression in each cell cluster. (B-C) t-SNE map of CD45^+^ cell clusters from tumor tissue.

**Figure S3. Characteristics of immune cell subsets.**
(A) Heat map of marker gene expression of immune cells. (B) Highlighted t-SNE plot of representative genes of immune cells.

**Figure S4.** **Analysis of differential gene expression and functional enrichment in the glioma immune microenvironment after treatment.**

(A-C) Comparison of differential gene expression analysis. PBS vs M0-EVs (A). PBS vs M1-EVs (B). M0-EVs vs M1-EVs (C). 0-6 represent B cells, granulocytes, macrophages, microglia, monocytes, NK cells, T cells. (D-F) Functional enrichment analysis of differentially expressed genes in glioma tissue after the indicated treatments. PBS vs M0-EVs (D). PBS vs M1-EVs (E). M0-EVs vs M1-EVs (F).

**Figure S5. Basic information of macrophage cell subsets among different groups.**

(A) Single-cell RNA sequencing t-SNE map of macrophage in the tumor. (B) the t-SNE map of anti-tumor and pro-tumor macrophage after the indicated treatments. (C) Heat map of marker gene expression of anti-tumor and pro-tumor macrophage. (D-G) Highlighted t-SNE plot of representative genes CD68 (D); Nos2 (E); CD40 (F), and CCL24 (G) of macrophages after treated with PBS, M0-EVs, or M1-EVs.

**Figure S6. *In vivo* antitumor efficacy of EVs and characterizations of engineered EVs.**

(A) Experimental design for evaluating the efficiency of tumor inhibition upon treatments with PBS, M0-EVs, and M1-EVs in GL261-IL13Rα2-luc tumor-bearing model mice. (B) Representative bioluminescence images of GL261-IL13Rα2-luc tumor-bearing mice after *i.v.* injection with different groups at the indicated time points. The blank area means mice were dead. (C) Corresponding quantification of the total flux in luciferase signals from panel (B) (*n* = 6). (D) Plasmid map of pSFFV-CAR (hIL-13Rα2)-SIRPα variant. (E) Western blotting analysis of CAR, SIRPα, and Alix in CS‑EVs and CS‑EVs treated with proteinase K. (F) Size and zeta potential changes of M1-C-EVs, M0-CS-EVs, and M1-CS-EVs in PBS over 8 days (*n* = 3). (G) TEM images of EVs after 8 days of storage. Scale bars, 100 nm. (H) Hydrodynamic diameter and zeta potential of M1-C-EVs, M0-CS-EVs, and M1-CS-EVs measured by DLS in 10% serum over 48 h (*n* = 3). All data are expressed as mean ± S.E.M.

**Figure S7. safety profile of engineered EVs.**

(A) SDS-PAGE analysis of M1-C-EVs, M0-CS-EVs, and M1-CS-EVs. (B) Cell viability of macrophages, Jurkat cells, dendritic cells, and GL261 glioma cells treated with EVs at concentrations ranging from 0 to 100 μg/ml (*n* = 6). (C) Serum levels of TNF-α and IL-6 at 4 h post-injection in mice treated with PBS or M1-EVs (*n* = 3). (D) H&E-stained slice images of major organs (heart, liver, spleen, lung, and kidney) of healthy C57BL/6 mice after *i.v.* injection of PBS, EVs, M1-C- EVs, M0-CS-EVs, and M1-CS-EVs. Scale bar, 100 μm. (E) Blood biochemistry test of healthy C57BL/6 mice after *i.v.* injection of PBS, EVs, M1-C- EVs, M0-CS-EVs, and M1-CS-EVs (*n* = 5). ALT: alanine transaminase; AST: aspartate aminotransferase; ALB: Albumin; ALP: alkaline phosphatase; UREA: urea. Data are presented as mean ± S.E.M.

**Figure S8. Systemic hematological effects of intravenously injected EVs in mice.**

Complete blood test of healthy C57BL/6 mice after *i.v.* injection of PBS, EVs, M1-C-EVs, M0-CS-EVs, and M1-CS-EVs. WBC: white blood cell; Lym: Lymphocyte; Mon: monocytes; Gran: Granulocyte; RBC: red blood cell; HGB: hemoglobin; HCT: hematocrit; MCV: mean corpuscular volume; MCH: mean corpuscular hemoglobin; MCHC: mean corpuscular hemoglobin concentration; RDW: red blood cell distribution width; PLT: platelets; MPV: mean platelet volume; PDW: platelet distribution width; PCT: packed cell volume.

**Figure S9. Characterization of the antitumor immune microenvironment upon CD47-SIRPα blockade.**

(A) Flow cytometry and (B) immunofluorescence imaging of IL13Rα2 expression on original and engineered GL261 cells. Scale bar, 10 μm. (C and D) *In vivo* fluorescence imaging and their quantitative analysis of major organs in GL261-IL13Rα2 tumor-bearing mice with *i.v.* injection of EVs and C-EVs. (E) Immunofluorescence staining and (F) positive area ratio of CD47 in GL261-IL13Rα2 tumor-bearing brain, dotted lines indicate tumor boundary (*n* = 3). Scale bar, 100 µm. (G) Representative images of U‑bottom plates after incubation of RBCs with EVs or S‑EVs at indicated concentrations (0-100 µg/mL). (H) Flow cytometry analysis of DiO‑labeled EV or S‑EV binding to mouse RBCs at 100 µg/mL (*n* = 4). (I-K) t-SNE plots highlighting expression of representative genes CD83 (I), S100A9 (J), and S100A8 (K) at single-cell resolution. (L) Immunofluorescence staining of CD8^+^ T cells in tumor-bearing brain after *i.v.* injection with PBS, S-EVs, or CS-EVs. Scale bars: 100 µm. Statistical analysis was performed by unpaired two-tailed t test (D). Data are presented as mean ± S.E.M. ****P* < 0.001.

**Figure S10. Histological safety and tumor immune phenotype after therapy.**

(A) H&E staining images of major organs (heart, liver, spleen, lung, and kidney) of GL261-IL13Rα2 tumor-bearing mice in each group at the end of therapy. Scale bar,100 µm. (B-D) Immunofluorescence staining for CD8 (B), CD86 (C), and CD206 (D) in tumor tissues from GL261-IL13Rα2 tumor-bearing mice after different treatments. Scale bar, 100 µm.

**Figure S11. Gating strategies for flow cytometric analysis of tumor-infiltrating immune cells.**

(A) Gating strategy to sort Ly-6C^+^ monocyte in tumor tissues gating on CD11b^+^CD45^+^ cells, as well as CD206^+^ pro-tumor and CD80^+^ anti-tumor macrophages in tumor tissues gating on F4/80^+^CD11b^+^CD45^+^ cells. (B) Gating strategy to sort IL13Rα2^+^ in tumor tissues gating on F4/80^+^CD11b^+^CD45^+^ cells. (C) Gating strategy to sort CD8^+^ and CD4^+^ T cells in tumor tissues gating on CD3^+^CD45^+^ cells. (D) Gating strategy to sort CD80^+^CD86^+^ dendritic cells in tumor tissues gating on CD11c^+^CD45^+^ cells.

**Figure S12. *In vitro* and *in vivo* assessment of targeted EV delivery efficiency in U87MG tumors.**

(A) Flow cytometry of IL13Rα2 expression on U87MG cells. (B) CLSM images of U87MG cells incubated with EVs and C-EVs. Scale bar, 20 μm. (C) Degree of cellular uptake of EVs and C-EVs in U87MG cells quantified by flow cytometry. (D to F) *In vivo* fluorescence imaging and their quantitative analysis of U87MG tumor-bearing mice with *i.v.* injection of EVs and C-EVs. (G) Immunofluorescence staining of the U87MG tumor-bearing mice after *i.v.* injection of EVs and C-EVs. Scale bar, 50 μm. (H and I) *In vivo* fluorescence imaging and their quantitative analysis of major organs in U87MG tumor-bearing mice with *i.v.* injection of EVs and C-EVs. Statistical analysis was performed by unpaired two-tailed t test. Data are presented as mean ± S.E.M. (*n* = 3). ***P* < 0.01, ****P* < 0.001.

**Figure S13.** **Validation of the antitumor efficacy and immune modulation of engineered EVs in U87MG glioma model.**

(A) Representative bioluminescence images of U87MG-luc tumor-bearing mice after *i.v.* injection with different groups at the indicated time points. The blank area means mice were dead. (B) Corresponding quantification of the total flux in luciferase signals from panel (A). (C) Survival rate of the tumor-bearing mice upon treated with different groups (*n* = 6). (D) Flow cytometric analysis of IL13Rα2^+^ in tumor tissues gating on F4/80^+^CD11b^+^CD45^+^ cells. (E) Anti-tumor macrophages (CD80^+^F4/80^+^) and (F) pro-tumor macrophages (CD206^+^F4/80^+^) in tumor tissues from U87MG tumor-bearing mice gating on CD11b^+^CD45^+^ cells (*n* = 5). Statistical analysis was performed by two-way ANOVA with a Tukey’s test (B), log-rank (Mantel-Cox) test (C), or one-way ANOVA with Tukey’s multiple comparisons tests (D-F). Data are presented as mean ± S.E.M. **P* < 0.05, ***P* < 0.01, ****P* < 0.001, *****P* < 0.0001

**Figure S14. Validation of EV targeting specificity toward EGFRvIII in CT-2A glioma model.**

(A) Flow cytometry of EGFRvIII expression on wild type (WT) and engineered CT-2A cells. (B-C) CLSM images of CT-2A (B) and CT-2A-EGFRvIII (C) cells incubated with EVs and C-EVs. Scale bar, 20 μm. (D) Degree of cellular uptake of EVs and C-EVs in CT-2A and CT-2A-EGFRvIII quantified by flow cytometry. (E) Immunofluorescence staining of the CT-2A-EGFRvIII tumor-bearing mice after *i.v.* injection of EVs and C-EVs. Scale bar, 50 μm.
